# Nicotine dependence and associated factors among persons who use electronic e-cigarettes in Malaysia - an online survey

**DOI:** 10.1186/s13011-023-00558-7

**Published:** 2023-08-29

**Authors:** Chandrashekhar T. Sreeramareddy, Sameeha Misriya Shroff, Shilpa Gunjal

**Affiliations:** 1 Division of Community Medicine, International Medical University, Kuala Lumpur, Malaysia; 2 School of Postgraduate Studies, International Medical University, Kuala Lumpur, Malaysia; 3 Clinical Oral Health Sciences, School of Dentistry, International Medical University, Kuala Lumpur, Malaysia

**Keywords:** Electronic cigarettes, Epidemiology. Nicotine, Dependence, Side effects

## Abstract

**Background:**

Nicotine dependence, factors associated with dependence, and self-reported side effects among people who use e-cigarettes are scarce in developing countries.

**Methods:**

A sample of 302 persons who currently use e-cigarettes was recruited from discussion forums on Reddit, Facebook, and the forum ‘lowyat’. The online Google form survey collected data on demographics, e-cigarette use, and the reasons, for cigarette smoking, Fagerstorm Test for Nicotine Dependence adapted for e-cigarettes (eFTND), and side effects experienced.

**Results:**

The mean age was 25.5 years (6.5), 60.6% were males and 86% had higher education. About 47% were using e-cigarettes only, 27.8% were currently using dual products (both electronic and conventional cigarettes), and 25.2% had also smoked cigarettes in the past. ‘*Less harmful than cigarettes’* (56.3%), ‘*because I enjoy it’* (46.7%), and ‘*it has a variety of flavors* (40.4%) were the common reasons for e-cigarette use. The mean eFTND score was 3.9 (SD = 2.2), with a median of four side effects (IQR 3–6), sore or dry mouth/throat (41.4%), cough 33.4%, headache (20.5%), dizziness (16.2%) were commonly reported side effects. eFTND score and side effects were higher among persons using dual products. By multiple linear regression analysis, males (β = 0.56 95% CI 0.45, 1.05, p = 0.033), dual-use (β = 0.95 95% CI 0.34, 1.56, p < 0.003), and use of nicotine-containing e-cigarettes (β = 0.66 95% CI 0.07, 1.25 p = 0.024) had higher eFTND score.

**Conclusion:**

Our findings of the study call for the placement of disclaimers about possible nicotine addiction and side effects of e-cigarette products.

## Background

Electronic cigarettes also known as “e-cigs,” “vapes,” “e-hookahs,” “vape pens,” and “electronic nicotine delivery systems (ENDS)” and electronic non-nicotine delivery systems (ENNDS) [1] have an estimated 68 million users worldwide in the year 2020 [2]. with projected sales of e-cigarette products expected to reach $40 billion by the year 2023 [3]. E-cigarette use is higher among youth and young adults in high-income and middle-income countries [4, 5]. E-cigarettes are promoted as harm-reduction products, as aids in quitting conventional cigarettes, and safer alternatives to conventional cigarettes are effective to quit conventional cigarettes as nicotine-containing. Indeed literature shows that e-cigarettes may improve quit rates compared with nicotine replacement therapy [6]. However, studies on reasons to use e-cigarettes have shown that persons who used dual products (both e-cigarettes and cigarettes) were using e-cigarettes mainly to reduce cigarette consumption, and to quit smoking [7] while the youth use e-cigarettes due to peer pressure, being attracted to the variety of flavors and the perceptions that e-cigarettes are less harmful than conventional cigarettes [8]. Reasons for the e-cigarette use, have regulatory implications, but understanding the intensity e-cigarettes or dependence on nicotine and self-reported adverse events by the persons who use e-cigarette is also important for the development of regulatory policies [9].

Nicotine concentration in some e-cigarettes is comparable to nicotine replacement drugs [10] but the rate of its’ release to the brain is slower than nicotine in conventional cigarettes [11]. Therefore e-cigarettes are thought to be less addictive than conventional cigarettes [12–15]. Duration of use, nicotine content are likely drivers of dependence and their long-term use leading to adverse health outcomes. The information about dependence, and side effects are needed to implement regulations such as display of labels on the devices and liquids [16]. Nicotine dependence among e-cigarette users measured with various instruments such as Hooked on Nicotine Checklist [17], Fagerstorm test for nicotine dependence (FTND) adapted for e-cigarettes [18], Penn State Dependence Index [19], E-Cigarette Addiction Severity Index (EASI) [20] have shown the presence of nicotine dependence. Therefore, the labels should contain disclaimers about nicotine content, its addictive potential and possible side effects of e-cigarette use. Such disclaimers are expected to deter the e-cigarettes users. Considering the currently available evidence about the health effects of e-cigarette [21, 22], it is imperative to study self-reported side effects experienced by persons who use e-cigarettes.

Malaysia has a big e-cigarette market with a prevalence of daily use at 5.8% in 2020 among adults and past 30-day e-cigarette use at 9.1% among youth aged 13–19 years [23]. The reasons for the use of e-cigarette among Malaysian university students [24], and perceptions of long term e-cigarette users who are active online site Malaysian Organisation of Vape Entity (MOVE) [25] have been reported. Another study has reported the reasons for e-cigarette use among cigarette smokers [26]. The common reasons cited were ‘*to reduce number of cigarettes smoked’*, ‘*assist in quitting cigarette smoking*’, and ‘*enjoyment of pleasant taste’*. However, reasons for e-cigarette use may vary by age and cigarette smoking status [8, 27]. Nicotine content and level are falsely reported on the labels on liquids available in Malaysia market [16, 28]. This raises a concern that the actual content of nicotine could be much higher than that indicated on the labels, perhaps a strategy to get the EC users addicted to nicotine. A study among university students has reported dizziness, and headache as side effects after using e-cigarette [24]. However, the self-reported adverse effects, nicotine dependence levels, and the reasons for e-cigarette use among those who also currently smoke conventional cigarettes and smoked cigarette in the past have not been studied in Malaysia. Considering the reports about the discrepancy between nicotine content displayed on the e-cigarettes and actual content it is imperative to study nicotine dependence among Malaysians who use e-cigarettes [16, 28]. Further, nicotine dependence using an instrument that is comparable to FTND and comparison of nicotine dependence according to exclusive e-cigarette use, dual product use and past cigarette smoking, and factors that determine the nicotine dependence among persons who use e-cigarettes are seldom reported in literature. Thus, we aimed to assess reasons for e-cigarette use, nicotine dependence, side effects, among persons who use e-cigarettes and the factors associated with nicotine dependence.

## Methods

### Ethical considerations

#### Ethical approval

was obtained from the International Medical University Joint Committee for Research and Ethics (MSPH I-2022(07). The online discussion group platform moderators on the social media and the study participants were informed about the purpose on a participant information sheet at the beginning of the survey questionnaire to obtain consent to participate. The participants were free to discontinue the survey anytime.

### Study design

A cross-sectional online questionnaire survey was conducted among persons who were using e-cigarettes. They were recruited online from the e-cigarette related discussion communities and platforms on social media.

### Sampling and sample size estimation

A convenience sampling strategy was used to select persons who were using e-cigarettes. Social media platforms such as Reddit, Facebook, and Forum ‘Lowyat’ having an estimated total number of users ranging from 1000 to 283,00 were selected. On these social media nine online Malaysian e-cigarette communities and discussion platforms were identified. Based on previous research on the standard deviation of 2.6 for eFTND score among all persons who use e-cigarettes [29] we anticipated a higher dispersion of eFTND score in a diverse group of persons using e-cigarettes i.e. using only e-cigarettes, dual products, and those who smoked conventional cigarettes in the past. We used sample size calculation for estimation of mean using the formula N=(Zσ/E)^2^ [30]. For a standard deviation of eFTND score of 4.5, 95% confidence limits, and precision of 0.5, the required sample size was 315.

### Study instrument

A structured close-ended English language questionnaire was designed to collect information under seven sections. Section one contained information related to sociodemographic characteristics such as age, gender, nationality, ethnicity, occupation, marital and education status, household income [(In Malaysian Ringgits (MYR), 1 USD = 4.5 MYR] and number of family members who smoke conventional cigarettes and e-cigarettes. Section two had information on smoking-related information such as the age at smoking initiation, duration, and frequency (daily or non-daily or not at all) of smoking cigarettes adapted from GATS [31]. Section three inquired about participants’ e-cigarette use-related information current use of e-cigarettes (daily, non-daily or not at all) such as duration, frequency, usage of flavored e-cigarettes, awareness about nicotine content in e-cigarettes, and reasons for the use of e-cigarettes. Section four inquired about the method of e-cigarette purchase, questions about introduction to e-cigarettes, source of online information related to e-cigarettes, and exposure to e-cigarette-related online advertisements. Section five obtained information about nicotine dependence through a modified FTND (Fagerstrom Test for Nicotine Dependence) questionnaire which was adapted from Rahman et al. 2020 [32]. Section six inquired about reasons for the use of e-cigarettes and side effects with an the option to choose multiple responses.

### Main outcome variables

#### Nicotine dependence

The eFTND score was the main dependent variable indicating the level of addiction to e-cigarette use. The eFTND test includes the original six items adapted to e-cigarette use by making minor changes such as replacing “cigarettes” with “e-cigarettes” [33]. Modified eFTND as a valid and reliable instrument (I-CVI value = 0.95 and test-retest reliability Spearman’s rank correlation coefficient value = 0.730) that can be applied to e-cigarette related studies [32]. Four items are binary questions that scored 1 and 0 points, and two items are multiple-choice questions that scored from 0 to 3 points. The total score can range from 0 to 10 points with 0 indicating the lowest level of e-cigarette use dependence and 10 indicating the highest level of nicotine dependence.

#### Reasons for e-cigarette use and self-reported side effects

Based on the literature 10 questions on reasons for e-cigarette use with each question having an option to select ‘yes’ or ‘no’ [24–26]. The participants were asked “Have you experienced any of the following side effects from using e-cigarettes?” A list of 14 side effects based on the previous literature [34, 35] was provided the participants indicated to check if they had ever experienced them.

### Predictor variables

Sociodemographic characteristics were age, sex, nationality, ethnicity, employment status, marital status, education status, and household monthly income. Tobacco use-related factors cigarette smoking status, the daily number of puffs e-cigarettes consumed, and awareness about nicotine content in e-cigarettes. The other factors such as introduction to e-cigarettes, online sources of information about e-cigarettes, and exposure to online e-cigarette-related advertisements.

### Data collection

Before distributing the online google form questionnaire, the instrument was pilot tested on 25 participants who were not part of the main study. These participants were selected through snowball sampling and google forms were used to design and display the information among persons using e-cigarettes. The pilot study aided in gathering feedback and detailed information on participants’ comprehension of the survey questions and ease of use. The researcher registered to join online communities and social media platforms on Reddit, Facebook, and the ‘Lowyat’ forum and sought permission from the moderators to share the survey link. Following approval, the google form questionnaire link was shared to the members. Only a few online platforms allowed users to share content without needing permission. On those platforms, the questionnaire was shared immediately. To avoid spam, the number of times the survey link could be reposted was limited to twice a week and thrice a week for two Reddit communities respectively, and once a week for the other seven platforms.

### Data analyses

Data was cleaned, coded, and transferred from Microsoft excel to SPSS version 27.0 for data analysis. Data was checked for normality. Descriptive statistics of sociodemographic characteristics of the study participants and the reasons for e-cigarette use were calculated. eFTND score, the number of side effects and the main reason for e-cigarette use were compared by cigarette smoking status. Appropriate statistical tests significance was used for these bivariate comparisons. The association between eFTND score and sociodemographic, and tobacco-related factors was tested by simple linear regression followed by multiple linear regression. Variables significant in simple linear regression were entered into multivariable linear regression model. Unstandardized beta coefficients and their corresponding 95% confidence intervals (95%CI) and standardised coefficients were estimated. Collinearity between the independent variable was tested using tolerance and variance inflation factor. Statistical significance was set at p < 0.05.

## Results

### Demographic characteristics of the respondents

A total of 302 persons who use e-cigarette completed survey. The description of demographics of persons who use e-cigarettes, and the e-cigarette and cigarette smoking behaviours are shown in Table 1. The median age was 18 years (IQR 20–31), 60.6% were males, 61.9% were single, 45.4% were students. Ethic distribution was roughly divided between Malay, Chinese and Indian i.e., 30% each. Majority of them (89%) of them were from families whose monthly income was over 6000 Malaysian ringgits (Table 1).

**Table 1:** Demographic characteristics and e-cigarette use behaviors of the survey respondents

Sociodemographics	n (%)	Tobacco use behaviors	n (%)
Age (years) mean = 25.5 (SD = 6.5)		Current e-cigarette and smoking status	
Sex		Exclusive e-cigarette use	142 (47.0)
Female	119 (39.4)	Dual user	84 (27.8)
Male	183 (60.6)	Current e-cigarette use/ past smoking	76 (25.2)
Ethnicity		Daily e-cigarette	
Malay	85 (28.1)	Daily	185 (61.3)
Chinese	93 (30.8)	Non-daily	117 (38.7)
Indian	84 (27.8)		
Others	40 (13.2)		
Marital Status		Use of Nicotine e-cigarette	
Married/ in a relationship	115 (38.1)	I do not know	60 (19.9)
Single/ widowed/ divorced	187 (61.9)	No	64 (21.2)
Employment Status		Yes	178 (58.9)
Student	137 (45.4)		
Employed	127 (42.1)		
Currently unemployed	38 (12.6)		
Monthly Household Income		Number of puffs per day	
Less than 6,000 RM	63 (20.9)	< 20	94 (31.1)
6,000 RM – 9,999 RM	131 (43.4)	20–50	126 (41.7)
10,000 RM and above	108 (35.8)	> 50	82 (27.2)
Introduction to e-cigarettes		Duration of e-cigarette	
Friends/ family/ peers	194 (64.2)	< 6 months	60 (19.9)
Internet/ social media	87 (28.8)	6 months-1 year	72 (23.8)
Health professionals	21 (7.0)	1–2 years	61 (20.2)
Source of online information		> 2 years	109 (36.1)
Online vape stores	91 (30.1)		
YouTube and others	71 (23.5)	At least one family member smokes cigarettes	60 (19.9)
Social media	101 (33.4)	At least one family member uses e-cigarettes	97 (32.1)
Health-related websites	39 (12.9)		
Online advertisement exposure			
No	205 (67.9)		
Yes	97 (32.1)		

### Cigarettes smoking and e-cigarette-related behaviors

Of the 302 persons who reported using e-cigarettes, 84 (27.8%) also reported of there were also smoking cigarette while 76 (25.2%) had smoked cigarettes in the past. This accounts for 142 (47%) persons who reported of exclusive e-cigarette use, 84 (27.8%) persons who reported of dual products use, and 76 (25.2%) had smoked conventional cigarettes in the past. Of those who reported that they were currently smoked cigarettes, 48 (15.9%) smoked cigarettes daily and 100 (33.1%) did not smoke cigarettes daily (Table 1). Of the persons who currently use e-cigarette, 117 (38.7%) did not use daily while 185 (61.3%) used e-cigarettes daily, more than a third of them had reported of initiating e-cigarette more than two years ago, over 40% of them had taken 20–50 puffs in a daily and about 58.9% of them reported that knew that e-cigarettes contain nicotine (Table 1). E-cigarettes were introduced by friends/peers to more than half of the respondents, online stores, and social media including YouTube were the most common source of information about e-cigarettes. Of the 302 respondents, 19.9% reported of at least one family member who were currently smoking cigarettes and 32.1% of them had at least one family member who were currently using e-cigarettes (Table 1).

### Reasons to use e-cigarettes

**Fig. 1:**
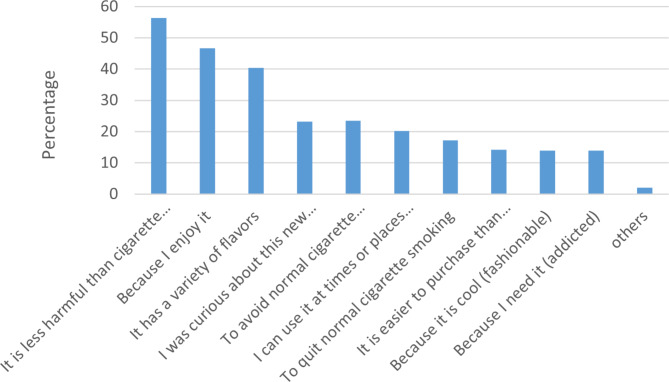
Distribution of reasons for using e-cigarettes

**Fig. 2:**
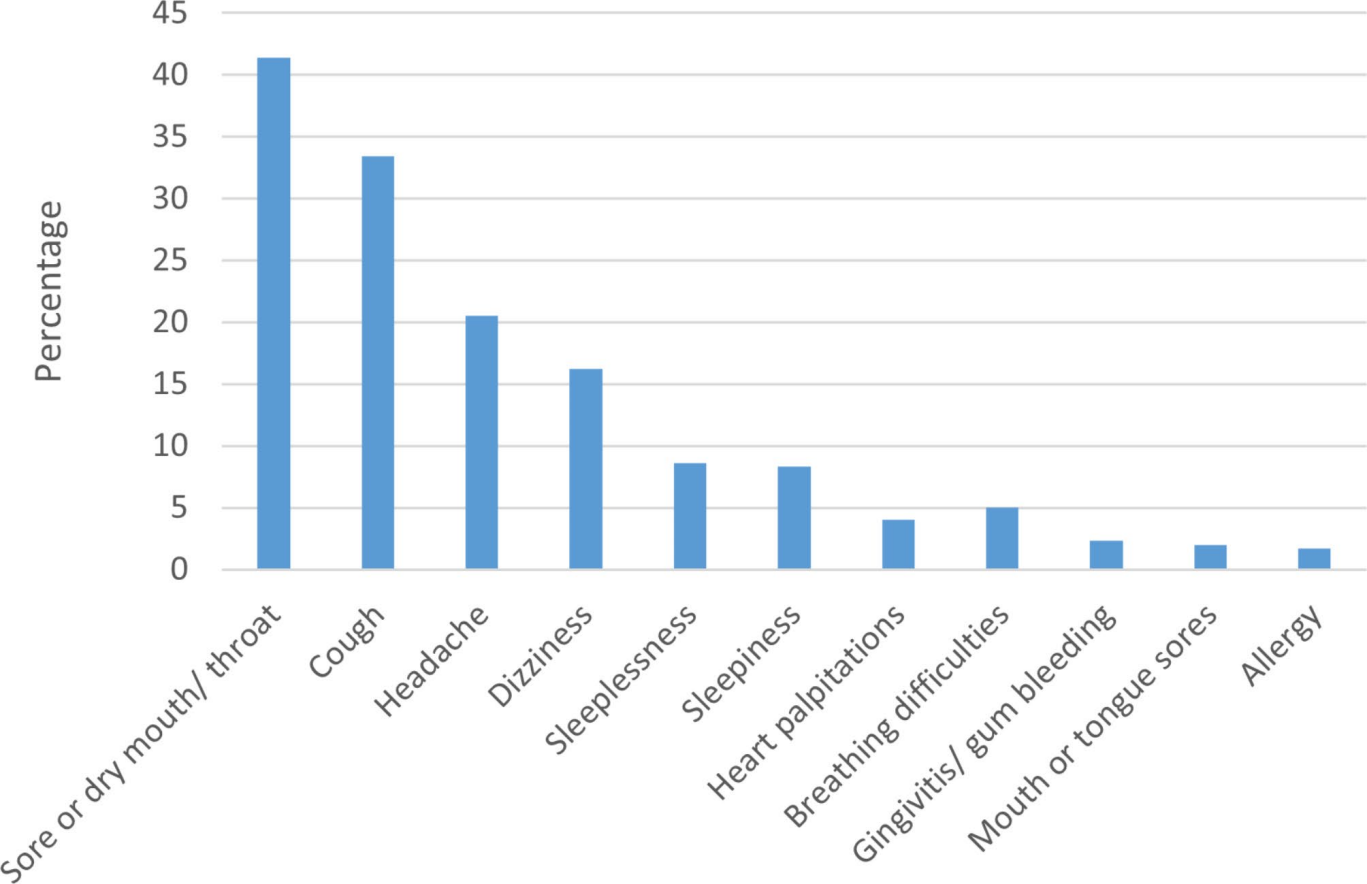
Distribution of self-reported side effects of e-cigarette

Frequency of reasons cited for use of e-cigarettes and side effects are shown in Figs. 1 and 2 respectively whereas the comparison of eFTND score, side effects and reasons according to smoking status are shown in Table 2. Among all the reasons for e-cigarette use about half the persons who use e-cigarettes reported that “e*-cigarettes are less harmful than conventional cigarettes* “(56.3%). The other reasons cited were “*I enjoy it*” (46.7%), and “*variety of flavors*” (40.4%) whereas nearly a quarter of them reported that “*curiosity to try the new product*“ (23.2%), “*to avoid cigarette smoking* “(23.5%) “*places and “use at time and places when cigarette smoking was not allowed*“(20.2%) (Table 2). The main reason for e-cigarette use was compared among persons who reported exclusive e-cigarette use, dual products use, and those who had smoked cigarettes in the past. Persons who reported exlcusive e-cigarette use (142) cited safer alternatives (50.7%), and product appeal-related reasons(33.1%): persons who reporated of dual products use (84%) cited reducing or quitting smoking (56%) and circumventing smoke-free laws (20.2%) as the reasons: persons who smoked cigarettes in the past cited safer alternative (30.3%) and quit or reduce smoking (38.2%) as the main reasons for e-cigarette use. The comparisons were statistically significant (p < 0.001) (Table 2).

**Table 2:** Comparison of FTND score, number of side effects, and the main reasons for electronic cigarette use among those who use only e-cigarettes, dual products, and those who smoked cigarettes in the past

	Exclusive e-cigarette use	Dual use	E-cigarette use among those who smoked cigarettes in the past	Test statistic	p-value
eFTND score [mean(SD)]^×^	3 (1.6)	5.3 (2.5)	4.1 (2.1)	34.9	< 0.001
Number of side-effects reported [mean(SD)] ^¥^	4.1 (1.9)	4.9 (2.5)	3.6 (1.9)	8.6	0.014
Main reasons for e-cigarette use π				85.2	< 0.001
Safer alternative	72 (66.7)	13 (12)	23 (21.3)		
To reduce or quit smoking	11 (12.6)	47 (54)	29 (33.3)		
Circumvent smoke laws	12 (32.4)	17 (45.9)	8 (21.6)		
Product appeal	47 (67.1)	71(10)	16 (22.9)		

× independent samples one-way ANOVA-test; ¥ Kruskal-Wallis test; π chi-square test

All comparisons were statistically significant at p < 0.05

### eFTND score and side effects

The mean and median FTND score was 5 (SD = 2.2, IQR, 4–7). A total of 164 (54.3%) respondents scored between 1 and 3 (low dependence) on the eFTND scale and 89 (29.5%) of the respondents scored between 4 and 6 (moderate dependence). A total of 49 respondents scored between 7 and 10 (high dependence) on the eFTND. The median number of side effects reported was 4 (IQR 3–6) (mean = 4.1, SD = 2.1). The most common side effects reported were dry mouth (41.4%), cough (33.4%), and headache (20.5%) (Fig. 2). By types of e-cigarette use, person who use only e-cigarettes (mean 3.0, SD 1.6) had significantly lower FTND scores than those who use dual products (mean 5.3, SD 2.5) and those who had smoked cigarettes in the past (mean 4.1 SD 2.1) (p < 0.001). Similarly, by types of e-cigarettes use persons who exlcuively use e-cigarettes (mean 4.1, SD 1.9) reported significantly lower side effects than those who reporetd dual products use (mean 4.9, SD 2.5) and those smoked cigarettes in the past smokers (mean 3.6 SD 1.9) (p < 0.014) (Table 2).

### Factors associated with eFTND score

Results of simple and multiple linear regression analyses are shown in Tables 3 and 4 respectively. By simple linear regression analyses, eFTND score was associated with higher age (β = 0.12, 95% CI 0.09–0.16, p < 0.001), male sex (β = 1.67, 95% CI 1.19–2.15 p < 0.001), being married/relationship(β=-0.64, 95% CI -1.15—0.13, p < 0.014), family members using e-cigarettes(β=-0.26 95% CI -0.51 -0.02, p = 0.031), currently employed (β = 1.60, 95% CI 1.09–2.11 p < 0.001) past cigarette smoking (β = 1.08, 95% CI 0.52–1.64, p < 0.001), and currently smoking cigarettes as well as using an e-cigarette(β = 2.29 95% CI 1.74–2.83, p < 0.001), aware of nicotine presence in e-cigarettes(β = 2.04 95% CI 1.45–2.63, p < 0.001), introduced to e-cigarettes by online sources (β = 1.35, 95% CI 0.82–1.88 p < 0.001) and health professional(β = 2.41 95% CI 1.47–3.35, p < 0.001), the occurrence of side effects(β= -1.90 95% CI -2.37, -1.43, p < 0.001), daily use(β = 0.13 95% CI 0.01, 0.25, p < 0.001), and longer duration (> 2 years) of e-cigarette use(β = 2.40 95% CI 1.77–3.04 p < 0.001) (Table 3).

**Table 3:** Demographic, and tobacco use-related factors associated with eFTND score by simple linear regression analyses

Variable	eFTND score	Unstandardized coefficient (95% CI)	p-value
**Age (years)** Mean = 25 (Sd = 6)		0.12 [0.09, 0.16]	*<* 0.001
**sex**			
Female	2.9 (1.6)	1	
Male	4.6 (2.3)	1.67 [1.19, 2.15]	*<* 0.001
Ethnicity			
Malay	4.2 (2.2)	1	
Chinese	4.1 (2.3)	-0.09 [-0.75, 0.55]	0.766
Indian	3.6 (2.9)	-0.59 [-1.26, 0.07]	0.083
Others	3.8 (2.4)	-0.40 [-1.23, 0.43]	0.344
Marital status			
Married/ in a relationship	4.3 (2.4)	1	
Single/ widowed/ divorced	3.7 (2.1)	-0.64 [-1.15, − 0.133]	0.014
Employment status			
Student	3.2 (1.9)	1	
Employed	4.8 (2.4)	1.60 [1.09, 2.11]	*<* 0.001
Currently Unemployed	3.6 (1.6)	0.37 [-0.38, 1.12]	0.335
Monthly family income			
Less Than 6,000 Rm	4.2 (2.4)	1	
6,000 Rm – 9,999 Rm	4.1 (2.2)	-0.17 [-0.84, 0.49]	0.603
10,000 Rm and above	3.5 (2.2)	-0.67 [-1.36, 0.01]	0.055
Family members who smoke cigarettes		-0.02 [-0.20, 0.15]	0.787
Family members who use e-cigarettes		-0.26 [-0.51, -0.02]	0.031
Current smoking status			
Use only e-cigarettes	3.0 (1.6)		
Dual use	5.3 (2.6)	2.29 [1.74, 2.83]	*<* 0.001
Past smoke	4.1 (2.1)	1.08 [0.52, 1.64]	*<* 0.001
Nicotine presence			
No	2.6 (1.2)	1	
Yes	4.6 (2.3)	2.04 (1.45, 2.63)	< 0.001
I do not know	3.3 (2.0)	0.79 (0.06, 1.51)	0.034
Introduced to e-cigarettes			
Friends/ Family/ Peers	3.3 (1.9)		
Internet/ social media	4.7 (2.5)	1.35 [0.82, 1.88]	*<* 0.001
Health Professionals	5.8 (2.0)	2.41 [1.47, 3.35]	*<* 0.001
Source of online information			
Online vape stores	4.0 (2.2)		
YouTube and others	4.5 (2.6)	0.5 [-0.32, 1.33]	0.233
Social media	3.7 (2.1))	-0.30 [-0.92, -0.32]	0.348
Health-related websites	3.6 (2.2)	-0.39 [-1.08, 0.28]	0.255
Seen online advertisement			
No	3.7 (2.2)	1	
Yes	4.3 (2.3)	0.05 [-0.03, 1.04]	0.064
Number of side effects		0.13 [0.01, 0.25]	0.029
Number of puffs per day			
< 20	4.0 (2.7)	1	
20–50	3.2 (1.5)	0.12 (-0.56, 0.79)	0.729
> 100	4.9 (2.1)	0.25 (-0.44, 0.94)	0.481
Duration of use			
< 6 Months	2.7 (1.6)	1	
6 Months-1 Year	3.2 (1.7)	0.52 [-0.16, 1.21]	0.134
1–2 Years	3.9 (1.9)	1.20 [0.48, 1.92]	0.001
> 2 Years	5.1 (2.4)	2.40 [1.77, 3.04]	0.001
Frequency of e-cigarette use			
Daily	5.1 (2,3)	1	
Non-Daily	3.2 (1.8)	-1.90 [-2.37, -1.43]	0.001

**Table 4:** Demographic, and tobacco use-related factors associated with eFTND score by multiple linear regression analyses

Variable	Unstandardized coefficient (95% CI)	p-value	Standardized coefficient
**Age (years)** Mean = 25 (Sd = 6)	0.35 [-0.03, 1.00]	0.256	0.11
**sex**			
Female	1		
Male	0.56 [0.45, 1.05]	0.033	0.12
Marital status			
Married/ in a relationship	1		
Single/ widowed/ divorced	-0.17 [-0.66, 0.32]	0.487	-0.04
Employment status			
Student	1		
Employed	-0.26 [-1.06, 0.60]	0.528	-0.06
Currently Unemployed	0.04 [-0.86, 0.93]	0.934	0.01
Family members who use e-cigarettes	-0.20 [-0.42, 0.01]	0.065	-0.09
Current smoking status			
Use only e-cigarettes	1		
Dual use	0.95 [0.34, 1.56]	0.003	*0.19*
Past smoke	0.25 [-0.32, 0.82]	0.386	0.05
Nicotine presence			
No	1		
Yes	0.66 (0.07, 1.25)	0.024	-0.13
I do not know	0.69 (1.04, 1.34)	0.037	-0.01
Introduced to e-cigarettes			
Friends/ Family/ Peers	1		
Internet/ social media	0.35 [-0.17, 0.86]	0.186	0.07
Health Professionals	0.81 [-0.12, 1.73]	0.089	0.09
Number of side effects	0.15 [0.04, 0.26]	0.007	
Duration of use			
< 6 Months	1		
6 Months-1 Year	0.48 [-0.16, 1.12]	0.139	0.09
1–2 Years	0.81 [0.09, 1.53]	0.029	0.15
> 2 Years	0.98 [0.20, 1.75]	0.014	0.21
Frequency of e-cigarette use			
Daily	1		
Non-Daily	-0.91 [-1.38, -0.45]	< 0.001	-0.20

By multiple linear regression analyses, sex, e-cigarette use in the family, the occurrence of side effects, dual-use, awareness of nicotine in e-cigarettes, duration of e-cigarette use was associated with eFTND score (Table 4). eFTND score was 0.56 units (β = 0.56 95% CI 0.45–1.05 p = 0.03) higher among men compared to women, eFTND score increased by 0.13 units (β = 0.13 95% CI 0.01–0.25, p = 0.007) for a unit increase in the number of side effects. Similarly, the eFTND score was higher by 0.95 units (β = 0.95, 95% CI 0.34–1.56, p = 0.003) among persons who reported dual product use compared to persons who reported exclusively e-cigarettes use. eFTND score was 0.66 units (β = 0.66, 95% CI (0.07–1.25, p = 0.024) higher among those who reported using e-cigarettes that contain nicotine as compared those reported using e-cigarette without nicotine. eFTND score was 0.98 units (β = 0.98, 95% CI 0.20–1.75, p = 0.014) higher among those who reported e-cigarette use of more than 2 years compared to < 6 months of e-cigarette use. FTND score was 0.91 units (β= -0.91, 95% CI -1.38–0.45, p = 0.001) lower among those who do not use e-cigarette daily compared to those who use them daily.

## Discussion

Our convenient sample of persons who use e-cigarettes over half of who were males with a mean age of 25 years, and active on social media platforms. The surveyed person who reported e-cigarette use reported harm reduction, to avoid and quit cigarettes as the main reasons for using e-cigarettes. Nicotine dependence measured using eFTND was of low to moderate level. And eFTND score was associated with male sex, dual use, daily, longer and frequent of use of e-cigarettes. eFTND score was also associated with awareness about nicotine in the e-cigarette, number of side effects experienced. Nearly all persons who use e-cigarettes reported an experience of at least one side effect, while those who use dual products and had smoked cigarettes in the past reported more side effects.

Dependence is an important consequence of any nicotine-containing product. Dependence among persons who use e-cigarettes often leads to subsequent cigarette smoking initiation, particularly among the youth known as the ‘gateway phenomenon’ [36]. Though e-cigarettes are reported to be less addictive than conventional cigarettes [13–15, 37]. Studies reporting dependence among persons using e-cigarettes are not comparable since different methods were used to measure dependence and the convenient sample of persons who exclusively use e-cigarettes users were recruited online or compared to different groups of tobacco product users [15, 38, 39]. Low dependence among persons using only e-cigarettes is comparable to studies that measured dependence using a similar tool i.e. eFTND [40]. Nevertheless, as measured by eFTND, higher dependence was reported among highly educated young adults using e-liquids containing higher nicotine concentrations. In our study persons who reported exclusively e-cigarette use had a low dependence than those who were using dual products. These findings are supported by the review of available evidence [41]. Those who reported of dual products use would be consuming higher amount of nicotine that was present in both tobacco products. It has been argued that the original FTND used to measure dependence among conventional cigarette smokers has limited adaptability to measure dependence among those who use e-cigarettes. Even though nicotine dependence is common for both e-cigarette and conventional cigarettes, tools originally developed for conventional cigarettes may not predict e-cigarettes use emphasizing the need for product-specific tools [42].

The number and type of self-reported adverse effects reported by persons who use e-cigarettes were comparable to published literature [43, 44]. Dry mouth/throat, cough, headache dizziness were commonly reported in previous studies [43, 44]. Respiratory symptoms reported by persons who use e-cigarettes did not significantly vary by the type of device [45] and adverse events were commonly reported by all those using e-cigarettes irrespective of user characteristics [34]. However, we did not have sufficient information on these to perform sub-analyses. The reported side effects are also in agreement with those reported in the review of all available evidence [22]. There is very little evidence about long-term risks to the cardiovascular system, but dizziness, palpitations, and headache perhaps are suggestive of present of nicotine in the e-cigarettes [24]. The three frequent reasons for the use of e-cigarette namely less harmful (safe), ‘enjoy it’, and ‘many flavors’ are comparable to previous national-level survey reports from Malaysia [46–48]. However, the reasons varied by daily or non-daily e-cigarettes use in the ITC survey. While for daily use reasons cited were for reducing cigarette smoking and quitting smoking, for non-daily use reasons reported were curiosity and offered by someone [47]. The most cited reasons for e-cigarette use is suggestive of positive impressions created by marketing e-cigarette as safe, with a variety of flavors available in the market.

By multiple linear regression analyses being male, duration of e-cigarettes use, daily e-cigarettes use and dual-use, nicotine e-cigarettes, and the number of side effects were associated with eFTND score. Similar to the results our study literature about factors associated with dependence among e-cigarette users have found that nicotine dependence among e-cigarettes was associated with initiation of e-cigarette use at a younger age, e-cigarette use among friends and more recent use [20], as well frequency of use, concurrent cigarette smoking, use of nicotine e-liquids and higher nicotine concentrations [49]. However, instrument used to measure nicotine dependence was different from that used in our survey i.e., eFTND. A national survey from China that used FTND has reported a high level of dependence among cigarette smokers. The study also reported nicotine dependence was associated with smoking intensity and pack-years [50]. The association of eFTND score with daily use of e-cigarettes and duration is in our study is comparable to dependence on cigarette smoking in the Chinese study which used FTND [50]. In our study, men had higher eFTND scores than women but in the Chinese study the tobacco dependence was nearly the same despite the very high prevalence of cigarette smoking among men [50]. In our study after adjustment for other factors effects size was relatively small relative to the difference in eFTND scores between men and women. As dependence is caused by nicotine present in the e-cigarettes persons who reported the use of e-cigarette containing nicotine had a two points higher eFTND score than those reported to use cigarettes without nicotine. It is of importance to note that eFTND score was higher among persons using dual products, persons who had smoked in the past and were using e-cigarettes up on Health Professionals advice. Though these differences did not attain statistical significance in multiple linear regression, the findings suggest that persons who currently smoke cigarettes or who had smoked cigarettes in the past continue to be addicted to nicotine albeit to a lower extent than those who had never smoked conventional cigarettes.

### Limitations

Findings may not be generalizable given the use of a convenience sample recruited from online discussion communities. The online survey method did not provide an opportunity to verify the nicotine level in the body or to verify cigarette smoking by testing carbon monoxide levels. As persons who use e-cigarettes were recruited from online for a dedicated e-cigarette related discussion, we could not compare the nicotine dependence among persons who smoked only conventional cigarettes. The daily amount of e-cigarette consumption was based on the reported daily number of puffs consumed is subject to recall bias. We did not estimate the amount of nicotine present, and the types of e-cigarettes used. Perhaps it is, for this reason, the eFTND score was not associated with the number of puffs consumed. However, due to the diversity in types e-cigarette products, and variability in the amount of nicotine content in them, it is not possible to obtain an objective measure of e-cigarette consumption like the number of cigarettes smoked per day. Comparison of nicotine dependence between people who use only e-cigarettes and those who use dual products is limited by the lack of an objective metric for nicotine consumption for e-cigarettes.

## Conclusions

Our findings suggest a low or moderate level of self-reported nicotine dependence among the people who use e-cigarettes, conforming previous reports. Nicotine dependence was associated with male sex, cigarette smoking status, duration, and frequency of e-cigarette use. The main reasons for e-cigarette use and the number of side effects experienced varied according to cigarette smoking status. Our findings on nicotine dependence are to be confirmed in a larger population-based sample survey. Self-reported low to moderate nicotine dependence and side effects among people who use e-cigarettes call for regulatory policy on disclaimers about addiction and possible side effects to be placed on e-cigarette products.

## Data Availability

The datasets analyzed during the current study are not publicly available but available from the corresponding author on reasonable request.

## Abbreviations

ENDS: Electronic Nicotine Delivery Systems
ENNDS: Electronic Non-nicotine Delivery Systems
eFTND: e-cigarette Fagerstorm Test for Nicotine Dependence
MOVE: Malaysian Organisation of Vape Entity
